# Human 5–HT_4_ and 5–HT_7_ Receptor Splice Variants: Are they Important?

**DOI:** 10.2174/157015907782793621

**Published:** 2007-12

**Authors:** Ian M Coupar, Paul V Desmond, Helen R Irving

**Affiliations:** 1 Department of Pharmaceutical Biology, Victorian College of Pharmacy, Monash University, Parkville, Victoria 3052, Australia; 2 Department of Gastroenterology, St. Vincent’s Hospital, University of Melbourne, Fitzroy, Victoria 3065, Australia

**Keywords:** Serotonin receptors, GPCR receptor isoforms, GPCR receptor splice variants, GPCR interacting proteins, desensitisation, functional intestinal disorders, irritable bowel syndrome.

## Abstract

G-protein-coupled receptors (GPCRs), which are encoded by >300 genes in the human genome, are by far the largest class of targets for modern drugs. These macromolecules display inherent adaptability of function, which is partly due to the production of different forms of the receptor protein. These are commonly called ‘isoforms’ or ‘splice variants’ denoting the molecular process of their production/assembly. Not all GPCRs are expressed as splice variants, but certain subclasses of 5–HT receptors are for example, the 5–HT_4_ and 5–HT_7_ receptors. There are at least 11 human 5–HT_4_ and three h5–HT_7_ receptor splice variants. This review describestheir discoveries, nomenclature and structures. The discovery that particular splice variants are tissue specific (or prominent) has highlighted their potential as future drug targets. In particular, this review examines the functional relevance of different 5–HT_4_ and 5–HT_7_ receptor splice variants. Examples are given to illustrate that splice variants have differential modulatory influences on signalling processes. Differences in agonist potency and efficacies and also differences in desensitisation rates to 5–HT occur with both 5–HT_4_ and 5–HT_7_ receptor splice variants. The known and candidate signalling systems that allow for splice variant specific responses include GPCR interacting proteins (GIPs) and GPCR receptor kinases (GRKs) which are examined.Finally, the relevance of 5–HT receptor splice variants to clinical medicine and to the pharmaceutical industry is discussed.

## INTRODUCTION

G-protein-coupled receptors (GPCRs) are by far the largest class of targets for modern drugs. These macromolecules are encoded by >300 genes in the human genome. Once formed, they are transported and embedded in the cell-surface, where they take on their functions of detecting and responding to a diverse array of ligands. Numerous diseases and disorders have been linked to mutations and polymorphisms in GPCRs and in their natural states these receptors are the targets of an increasingly large number of therapeutic agents. It has been estimated that 50% of all modern drugs and almost one-quarter of the top 200 best-selling drugs in 2000 modulate GPCR activity (see [32] for review). Studies into GPCR splice variants or isoforms is a new research area that opens the possibilities to further refine safety margins of therapeutic drugs.

**Fig. (1):**
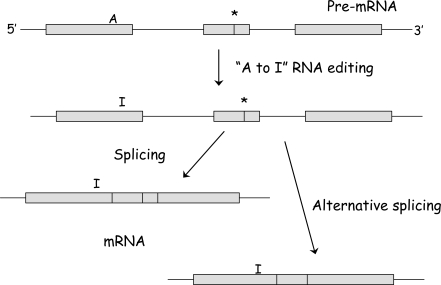
Methods of isoform or splice variant generation. RNA editing from adenosine deamination to form inosine which is read as guanosine and so codes for different amino acids. Alternatively if additional splice junctions are present (e.g. at * but also at the ends of exons depicted by boxes), exons can be incorporated or removed (drawn) resulting in different length mRNAs and so different proteins.

A general property of GPCRs is that they have inherent adaptability built into their function, which is partly due to the production of different forms of the receptor protein. Thus, different products can be generated from a single GPCR gene by the combination of alternative forms of particular exons. This process is referred to as ‘alternative splicing’ and translated products are called ‘splice variants’ or more commonly ‘isoforms’(see Fig. **1**). Over 70% of multi-exon genes expressed in humans are alternatively spliced to form various splice variants and the proteins involved in cellular communication are common amongst examples [41]. The discovery that particular splice variants are tissue specific (or prominent) has highlighted their potential as future drug targets. Therefore, just as the discovery of different receptor subgroups opened up vast opportunities to develop new drug treatments, the discovery of splice variants promises to further expand and refine these opportunities. Examples are to be found with prostaglandin EP_3_ receptors, which are subject to splice variance at the C-terminus and, to date, 10 splice variants have been identified across species, six of these being expressed in man. In addition, there is evidence for a splice variant form of EP_1_ receptor that lacks the highly conserved seventh transmembrane domain (TM). Amongst adrenoceptors, four splice variants of the α_1A_-AR have been reported as well as variants of the β_3_-AR. Of the serotonin (5-hydroxytryptamine, 5–HT) receptors, the 5–HT_4_ and 5–HT_7_ receptors in particular are noteworthy for the production of several splice variants [40].

Several questions arise when considering the purpose of these GPCR splice variants. Are they expressed in specific tissues? Do they modulate signalling processes in different fashions? If so, could they act as potential therapeutic targets? Here we will discuss these questions in relation to serotonin receptor splice variants with an emphasis on the distribution of 5–HT_4_ and 5–HT_7_ receptor splice variants and their potential as therapeutic targets. We will emphasise the neuropharmacological aspects. However, 5–HT receptors are widely distributed, so it is important to note that substantial information has been acquired about their splice variants by studying peripheral tissues. Although 5–HT receptor splice variants are localised to nerve elements in many tissues, others are found in effector cells, such as smooth muscle. It is also important to note, that much of our knowledge about the function of splice variants comes from cell culture techniques. Although these can be criticised for being artificial and minimalist, they are the only practical way of studying individual splice variants, because most native cells express a mixture. In this review we first outline a brief commentary on the functions and classification system of 5–HT receptors. We will also refer to some excellent and extensive reviews on other aspects of the subject which are not covered in this paper. For instance, the structural and operational characteristics of 5–HT receptors are described [38] and the distribution and function of 5–HT_7_ receptors has been reviewed [48,73]. There are also extensive reviews of the medicinal chemistry and pharmacology of 5–HT_4_ agonists and antagonists [46] and of 5–HT_7_ receptor ligands [33]. In addition, the clinical relevance of 5–HT receptors in pathophysiological conditions and their targeting with therapeutic drugs is covered [29].

## 5–HT

5–HT has diverse physiological effects and broadly these encompass regulation of the cardiovascular, digestive and central nervous systems. Mammalian physiologists had known since the 1800s that a vasoconstrictor substance was formed when blood was allowed to clot. In 1948, Rapport named the serum vasoconstrictor ‘serotonin’**and a year later he discovered that the active substance was 5-hydroxytryptamine. 5–HT is widely distributed in nature, occurring in both plants and animals. In mammals the largest amount of 5–HT is present along the length of the gastrointestinal tract (60-90%), mainly in enterochromaffin (EC) cells of the mucosal layer. The remainder occurs in the enteric nervous system with significant distribution in the brain and spinal cord, the heart and adrenocortical cells. 5–HT is synthesised from tryptophan in these sites, but the 5–HT content of platelets is acquired mainly from EC cells. It is now recognised that disturbances in the levels of 5–HT and/or the densities of its receptors contributes to the pathogenesis of many clinical conditions. Some examples are the carcinoid syndrome and gastrointestinal motility disorders in the periphery, and migraine, depression, anxiety, schizophrenia, obsessive compulsive disorders, eating disorders and the serotonin syndrome centrally.

## 5–HT RECEPTORS

The physiological and pathophysiological effects of 5–HT are mediated by at least 14 different receptor subtypes [2,37]. This relatively large number is attributed to the long evolutionary history of the 5–HT signalling system, which predates the separation of vertebrates and invertebrates, some 600 million years ago. Consequently, there has been abundant time for gene duplications, followed by mutations and sequence shifts to form the different genes encoding for the different subtypes. It has been speculated that the ancestral 5–HT receptors functioned to facilitate cell to cell connections and to promote growth and differentiation. This diversified to the level of complexity now apparent in the mammalian brain (for reviews, see [5,8,18,71]). 

All mammalian 5–HT receptors are members of the GPCR superfamily of membrane-bound receptors (also called metabotropic receptors) with the exception of the 5–HT_3_ receptor which is an ion channel. These are classified into seven distinct classes, or families, according to their structure, pharmacological properties and preferred effector mechanisms according to the current IUPHAR appellation of 5–HT_1_, 5–HT_2_, 5–HT_3_, 5–HT_4_, 5–ht_5_, 5–HT_6_ and 5–HT_7_. Some of these classes include multiple receptors, which share similar structural and effector properties, but display very different operational profiles. The lower case character denotes that the class concerned has not been ascribed functional roles although structural and transduction information is known. The 5–HT_1_ receptor class comprises five different receptors; 5–HT_1A_, 5–HT_1B_, 5–HT_1D_, 5–ht_1e_ and 5–HT_1F_ which couple preferentially to G_i_/G_o_ to inhibit cAMP formation. The 5–HT_2_ receptor class comprises three receptors 5–HT_2A_, 5–HT_2B_ and 5–HT_2C_ that couple preferentially to G_q_/G_11_ to increase the hydrolysis of inositol phosphates and elevate cytosolic [Ca^2+^]. Selective antagonists for each receptor are now becoming available. The 5–HT_3_ receptor is a pentameric ion channel that appears to be located exclusively in neuronal tissue where it mediates fast depolarization. 5–HT_4_, 5–HT_6_ and 5–HT_7_ receptors all couple preferentially to G_s_ and promote cAMP formation, while the 5–ht_5_, receptor is able to couple to several signalling pathways including G_i_/G_o_ [40]. Numerous selective 5–HT_4_ receptor agonists and antagonists are now available and selective antagonists for the 5–HT_7_ receptor and putative 5–HT_6_ receptor antagonists have also recently been reported. Many therapeutic drugs target 5–HT receptors, with notable examples being: the anxiolytic buspirone (5–HT_1A_ agonist), the antimigraine drug sumatriptan (5–HT_1B/D_ agonist), the antidepressant mianserine (5–HT_2A/C _antagonist), the antiemetic ondansetron (5–HT_3_ antagonist), and tegaserod (5–HT_4_ partial agonist), which has been used to treat the constipation-predominant form of irritable bowel syndrome (IBS). 

## 5–HT RECEPTORS WITH ISOFORMS

The 5–HT_1_ receptor family (5–HT_1A_, 5–HT_1B_, 5–HT_1D_, 5–ht_1e_ and 5–HT_1F_) are intronless so do not form splice variants [18,68]. Although the 5–HT_2_ receptor family (5–HT_2A_, 5–HT_2B_ and 5–HT_2C_) contains introns, the splice variants formed by alternative splicing produce truncated non-functional proteins. However, the 5–HT_2C_ receptor forms isoforms through RNA editing involving the enzyme family of adenosine deaminases that act on RNA (Fig. **1**) where agonist potency, activation of phospholipase C and selectivity of G protein coupling are generally reduced (for a review see [67]). The 5–HT_4, _5–ht_6_ and 5–HT_7_ receptors contain introns [18,68] and it has been demonstrated that the well characterised 5–HT_4_ and 5–HT_7_ receptors form multiple splice variants. These are the subject of this review.

### Human 5–HT_4_ Receptor Splice Variants

There are at least 11 human 5–HT_4_ receptor splice variants. The major relevance of this recent knowledge is that differences in the tissue distribution and function of 5–HT_4_ splice variants could potentially be used as a basis for new drug development. For instance, discovery of heart-selective drugs is achievable if the heart is found to express a 5–HT_4_ splice variant as the therapeutic target that is unique to the heart or more prevalent to the heart than other organs.

**Fig. (2):**
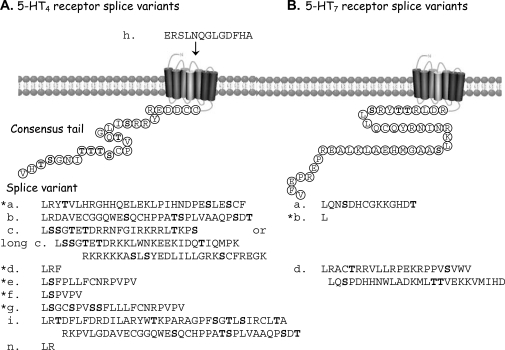
Serotonin receptor splice variants. **(A)**. The 11 human 5–HT_4_ receptor splice variants. Most of the splice variants occur in the C terminal tail following the splice site coding for the amino acid leucine 358 (shown as first amino acid in each splice variant). However, splice variant h5-HT_4(h)_ occurs in the second extracellular domain and has been reported to contain the “b” splice variant tail. **(B)**. The 3 human 5–HT_7_ receptor splice variants which occur in the C terminal tail following the splice site at coding for the amino acid leucine 432 (shown as first amino acid in each splice variant). Potential phosphorylation sites (S/T) are in bold; and * indicates a PDZ ligand.

The 5–HT_4_ receptor present in the human atrium was the first to be cloned and characterised. Following its discovery in 1997 and naming as h5–HT_4(a)_ [14]; several other splice variants have been cloned: h5–HT_4(b)_ [15,72], two different h5–HT_4(c)_ splice variants [13,15], h5–HT_4(d)_ [15], h5–HT_4(e)_ [13], h5–HT_4(f)_ [13], h5–HT_4(g)_ [13,24] (formerly called (e) [58] see [46]), h5–HT_4(i)_ [20], h5–HT_4(h)_ [13] the only example of an internal splice variant insert occurring in the 2^nd^ extracellular loop, and h5–HT_4(n)_ [74] (Fig. **2A**). From the viewpoint of drug discovery, the tissue distribution of these human splice variants shows a degree of specificity (see [46] for review). In addition, cellular studies show that desensitisation rates of 5–HT_4_ receptor splice variants depends upon the GPCR kinases (GRK) present [10] and the splice variant [64].

The h5–HT_4(b)_ is dominant in most tissues and the h5–HT_4(a)_ is also common, so opportunities for drug discovery need to take advantage of other tissue-specific splice variants. Table **1** summarises the current state of knowledge. Focussing on the atrial tissue of the heart, it can be seen that it is relatively well endowed with splice variants compared to the kidney and bladder. Also, it expresses the (n) splice variant, as does the brain and oesophagus, but this is absent from the kidney, bladder, stomach, ileum and colon. The current state of knowledge shows the human small intestine and colon also express h5–HT_4(d)_ and that this is not found in other tissues [13,15,56]. Many of the other splice variants are more widely distributed, so it is important to map both their distribution and quantity as their actual levels of expression may be low. 

Interestingly, 5–HT_4_ receptor splice variant expression has been shown to change dramatically in cancerous tissue. Normal adrenal tissue expressed both 5–HT_4(a)_ and 5–HT_4(b)_ and rarely 5–HT_4(d)_ splice variants while adrenocortical aldosterone producing adenomas increased their expression of 5–HT_4(d)_ and no longer expressed 5–HT_4(a)_ or 5–HT_4(b)_ receptor splice variants [22]. These results indicate the importance of knowing both receptor expression patterns in normal and diseased tissue as there is the potential to modulate receptor function with splice variant selective drugs.

### Human 5–HT_7_ Receptor Splice Variants

The 5–HT_7_ receptor was first cloned from human tissues in 1993 [7]. Three human 5–HT_7_ splice variants were then discovered and named, h5–HT_7(a)_, h5–HT_7(b)_ and h5–HT_7(d)_ that differ in their C terminal tails [36] (Fig.**2B**). It was found that the brain and spleen contained relatively small amounts of 5–HT_7(d)_ mRNA. However, h5–HT_7(d)_ was reported later to be predominantly expressed in the human small intestine and colon together with a certain amount of the h5–HT_7(d+5)_ fragment [43]. The known distribution of 5–HT_7_ receptor (or more prevalent) is summarised in Table **1**.

## THE C TERMINAL TAILS

All of the splice variants of both the h5–HT_4_ and h5–HT_7_ receptors (except h5–HT_4(h)_) differ in the sequences of their intracellular (C-terminus) tails, but share an identical sequence up to Leu 358 for h5–HT_4_ and Leu 432 for h5–HT_7_ (see Fig.**2**). This major portion contains the 7 transmembrane loops and the recognition site for 5–HT.

### Differences in Agonist Potency

The C-terminal tails of 5–HT_4_ receptor splice variants have been found to directly influence their functional properties and this is most dramatically seen in their transduction of agonist responses. A notable example is renzapride, which is nearly 20 times more potent at the h5–HT_4(d)_ than at the (g) splice variants in inducing cyclic AMP formation in COS cells. Another difference is that renzapride behaves as a full agonist at the h5–HT_4(d)_, but is a partial agonist at the (g) variant [59]. One interpretation for this phenomenon is that the C-terminus regions exert different torsion forces on the conserved transmembrane loops causing different steric presentations of the active site to its ligands. Some indirect support for this explanation comes from our observation that the different splice variants exhibit over 10-fold variations in their affinities for ligands in binding studies (Coupar, Tochon-Danguy, Irving unpublished observations). Another explanation for the functional differencesbetween 5–HT_4_ splice variants is that they can link to different G proteins. Experiments using human embryonic kidney (HEK) cells have shown that the potencies of 5–methoxytryptamine at 5–HT_4(a)_ and 5–HT_4(b)_ splice variants are different and that this is correlated to coupling to only G_s_ and to G_i/o_ plus G_s_ proteins, respectively [63]. More recent experiments using adenoviral expression of h5–HT_4(b)_ and 5–HT_4(d)_ splice variants in rodent cardiac myocytes that do not naturally express 5–HT_4_ receptors demonstrated that the 5–HT_4(d)_ receptor was more efficiently coupled to adenylyl cyclase [23]. In addition, it was shown that pertussis toxin potentiated the stimulatory effect of 5–HT on L–type Ca^2+^ current in rat myocytes expressing the 5–HT_4(b)_ splice variant but not the 5–HT_4(d)_ [23] providing further support for the suggestion that the (b) splice variant couples to both G_s_ and G_i/o _proteins.

5–HT_7_ receptors preferentially couple to adenylyl cyclase *via* G_s_α [1] similar to 5–HT_4_ receptors. The screening of four agonists and a larger set of antagonists has so far failed to show any differences in binding affinities, potencies or efficacies at the three h5–HT_7_ splice variants [43,44]. However, with the discovery of an increasing number of 5–HT_7_ ligands (see [33] for review) this situation may change. A recent study, also using HEK 293 cells, has shown that the h5–HT_7(d)_ splice variant exists in a greater internalised state in the absence of agonist (5–carboxamidotryptamine) compared to the other two variants; h5–HT_7(a) _and h5–HT_7(b)_. Interestingly, the 5–HT_7_ antagonist, SB-269970, induced a partial translocation of the h5–HT_7(d)_ variant from cytoplasm to plasma membrane. Another noted difference was that the h5–HT_7(d)_ variant was associated with a lesser efficacy at stimulating adenylyl cyclase. As a result, it was suggested that the C terminal tail of the h5–HT_7(d)_ splice variant, which is the longest of the three human splice variants, may contain a motif that interacts with cellular transport systems to limit the amount destined for the plasma membrane [34]. 

The ability of a ligand to provoke a GPCR-mediated response is measured in terms of ‘efficacy’ and the ligand is referred to as an ‘agonist’ in classical pharmacological terms. It is now apparent that a balance occurs between the molecular mechanisms controlling receptor signalling, desensitisation and resensitisation or down regulation. Hence, the selectivity of agonists may be influenced by differences in the individual rates at which their splice variants desensitise and/or interact with numerous intracellular GPCR interacting proteins (GIPs). 

### Differences in Intracellular Signalling Modulated by GIPs

The most common GIPs interact with the C terminal tails of GPCRs; and PDZ domain containing proteins are the most abundant members of this class. PDZ domains were first recognised as sequence repeats contained in three separate proteins: **P**ost synaptic density (PSD) protein PSD-95, **D**iscs large protein (the *Drosophila* homologue) and tight junction protein **Z**O-1. PDZ proteins are involved in scaffolding multi-protein complexes and have roles in protein trafficking (see [62] for a review). PDZ proteins bind to specific conserved consensus sequences that are found at the C-terminal end of proteins. These consensus ligands have been classified as class I PDZ ligand of S/TXǾ , class II of ǾXǾ and class III contains E/DXǾ where Ǿ represents a hydrophobic residue and X any amino acid [62]. Many GPCRs express a PDZ ligand at the extremity of their C terminus (usually the last 3-4 amino acids). Thus the C terminal tail of GPCRs can contain a PDZ ligand at its extremity while residues upstream of the last 3-4 are important in modulating the specificity of interactions with other proteins [17]. An example of how different splice variant tails can influence protein interactions is depicted in Fig. (**3**). This example is based on results of proteomic studies on mouse brain where two 5–HT_4_ receptor splice variants with different PDZ ligands in the extremity of their C terminal tails were used as a bait ligand to isolate the interacting proteins [42].

Several serotonin receptors contain PDZ ligands at the extremity of their C-terminal tails and have been the focus of investigation. GIPs containing PDZ domains specifically regulate receptors in the serotonin receptor family. The 5–HT_2A_ receptor contains a PDZ ligand at its extreme C terminus and directly binds to PSD-95 which augments signal transduction and inhibits agonist-induced receptor internalisation [75]. Moreover, recent proteomic experiments have demonstrated that the PDZ ligand of the 5–HT_2A_ receptor interacts with a different set of PDZ proteins to that of the 5–HT_2C_ receptor [12]. It is noteworthy that PSD-95 interacts with 5–HT_2A_ and 5–HT_2C_ but not 5–HT_4(a)_ receptors indicating that the different tails containing PDZ ligands interact with specific sets of proteins [12,42]. These specific sets of different PDZ proteins probably contribute to the different signal transduction properties of these receptors. Recently, another example of a GIP involved in serotonin receptor function has been reported where a small protein, p11, is involved in specifically transporting 5–HT_1B_ receptors to the plasma membrane [69]. These findings provide support to the tenet that “specific sets of GIPs interact with different sets of receptor splice variants and that this is also tissue dependent”. 

There are five 5–HT_4_ and one 5–HT_7_ receptor splice variants with canonical PDZ C–terminal extremity ligand binding sites (Fig. **2**). So far, there have been no reports on GIP interactions with any 5–HT_4_ or 5–HT_7_ receptor splice variants in human tissue. However, a recent study identified 13 GIPs (mainly PDZ domain proteins) that interact with either the mouse 5–HT_4(a)_ or 5–HT_4(e)_ receptors [42]. Of these proteins, 10 interact specifically with the mouse 5–HT_4(a)_ receptor splice variant. One of these is sorting nexin (SNX27) which is enriched in the brain and is involved in escorting the 5–HT_4(a)_ receptor splice variant to early endosomes for desensitisation. Another protein, NHERF appears to be involved in directing the 5–HT_4(a)_ receptor to the microvilli region where the two proteins are co-localised with another protein called ezrin [42] that interacts with the membrane phosphatidylinositol-(4,5)-bisphosphate [9]. The 5–HT_4(b)_ splice variant does not contain a PDZ domain (Fig. **2A**) and did not concentrate in the microvilli area. While the 5–HT_4(e)_ receptor splice variant co-localised with CIPP which is a scaffolding protein and importantly this was not seen with the 5–HT_4(b)_ receptor splice variant which does not contain a PDZ ligand [42]. These exciting results do indicate that the PDZ ligand domains of 5–HT_4_ or 5–HT_7_ receptor splice variants contribute to the receptor localisation and also suggest that rates of receptor desensitisation may vary with the splice variant expressed. 

### Differences in Desensitisation Rate

**Fig. (4):**
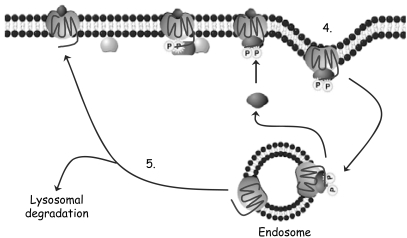
Process of GPCR desensitisation. **(1)**. Activation and dissociation of G protein. **(2)**. Gβγ recruits GPCR kinase (GRK) which phosphorylates the receptor. **(3)**. Attachment of arrestin to GPCR **(4)**. Internalisation of the arrestin – GPCR complex by endocytosis. **(5)**. Dephosphorylated GPCR returns to the cell surface (resensitisation) or is degraded in the lysosome (down regulation).

The diversity in C-terminus sequences probably also contributes to the well known pharmacological fact that 5–HT induces desensitisation (tachyphylaxis) at different rates and magnitudes in different tissues. This natural phenomenon functions to limit the biological response to endogenous substances, such as 5–HT by uncoupling the GPCR from its signal transduction pathway (for reviews, see [30,57]). Desensitisation generally begins with agonist-induced phosphorylation of the GPCR by GPCR receptor kinases (GRKs). The cytosolic proteins, arrestins then bind the phosphorylated GPCRs and prevent further coupling of that GPCR with G proteins and so reduce second messenger synthesis. The arrestin-GPCR complex is internalised by endocytosis where it can be recycled back to the membrane or degraded [30,57] (Fig. **4**). GRKs play a critical role in GPCR desensitisation. There are three GRK subfamilies: rhodopsin kinase containing GRK1 and 7; β adrenergic receptor kinase (GRK2 and 3); and GRK4 group (GRK4, 5 and 6). Both GRK2 and GRK5 can phosphorylate many GPCRs including 5–HT_4_ receptor splice variants [10] and GRK2 in particular is crucial for embryonic cardiac development [57]. The role of GRKs and receptor desensitisation has been the focus of many studies but their effects on serotonin receptors are less well documented. 

Desensitisation also has the potential to lessen the beneficial effects of agonists when used as therapeutic treatments. Indeed, we have established that desensitisation of native 5–HT_4_ receptors occurs *in vivo* in the rat. We found that 5 day infusions of 5–HT (75 μg/kg per hour) induced rightward shifts of the 5–_4_ receptor-mediated concentration-effect curves to 5–HT and the partial 5–HT_4_ agonist, SC 53116, in the oesophagus [55]. Therefore, knowledge of the extent and mechanisms of desensitisation in human tissue is crucial to the success of drug discovery programs aimed at developing 5–HT receptor agonists. In spite of this, only two studies have investigated the desensitisation properties of h5–HT_4_ receptor splice variants (human c and d variants [60]; mouse a, b, e, and f variants [10]) in cell culture. On the other hand, the h5–HT_7_ receptor splice variants all mediate heterologous desensitisation, which seems to be induced by both the agonist 5–HT and some antagonists [45]. In addition, all h5–HT_7_ receptor splice variants mediate heterologous desensitisation of endogenous Gs-coupled receptors in HEK 293 cells through unknown mechanisms that are independent of cAMP dependent protein kinase activation [3,45]. 

## CLINICAL RELEVANCE 

### 5–HT_4_ Receptors

More than 50 patents have been lodged since 2000 covering potential clinical applications for 5–HT_4_ agonists (ISI Web of Knowledge, Derwent Innovations Index, (http://portal.isiknowledge.com/portal.cgi)). The claims are mainly for treatments of the digestive tract, notably irritable bowel syndrome (IBS), but also for gastroesophageal reflux disease (GORD), delayed gastric emptying and chronic constipation. The main rationale for these applications is that 5–HT_4_ receptors are distributed along the length of the digestive tract, where their localisation on cholinergic nerves functions to enhance the release of acetylcholine. Localisation to smooth muscle cells also affects muscle tone. In the oesophagus, 5–HT_4_ receptors are thought to be located in the presynaptic nerve terminals of cholinergic interneurons and motor neurons [16], as well as the muscularis mucosa of the rat oesophagus, where they mediate relaxation directly [11]. In the intestines, 5–HT_4_ receptor activation was originally shown to enhance acetylcholine released in isolated preparations of the guinea-pig ileum [25-27]. Patents also cover treatment of CNS disorders, such as Alzheimer's disease, anorexia, drug dependence, migraine and pain. As in the digestive tract, 5–HT_4_ receptor function in the brain is to enhance the release of acetylcholine, but they also modulate the release of dopamine, GABA and 5–HT [8], hence the interest in 5–HT_4_ agonists for treating cognitive disorders. Encouraging results have been obtained in rats, where the 5–HT_4_ agonist, RS 67333, improved associative memory and the 5–HT_4_ antagonist, RS 67532, decreased memory [52]. Their potential in the treatment of anorexia is suggested from experiments using obese mice which showed that the 5–HT_4_ agonist, mosepride, improved food intake [4]. None of the patents claim the novel 5–HT_4_ receptor agonists are splice variant-selective, however, there are patents covering the DNA of splice variants themselves. One suggests gene therapy using the nucleic acid sequence of the 5–HT_4(h) _splice variant for gastrointestinal diseases, while another the 5–HT_4(d)_ splice variant for treating cardiac and bladder disorders. Reference to Table **1** shows there is potential for refining drug selectivity because the target organs for these conditions contain different combinations of splice variants. To date the digestive tract has received the greatest attention as an organ for treating various disorders using 5–HT_4_ agonists. Currently, these drugs are used to treat several types of motility disorders including gastro-oesophageal reflux disease (GORD). The most commonly used drugs were cisapride and metoclopramide, but cisapride has been virtually withdrawn (available on limited access) due to its ability to induce rare, but potentially fatal cardiac arrhythmias. Metoclopramide is a relatively old 5–HT_4_ agonist with significant affinity at other receptor types. The most selective 5–HT_4_ agonist to date is tegaserod, which is used to treat IBS. Consequently, the rational for using tegaserod for the treatment of IBS is outlined next.

### Irritable Bowel Syndrome

It has been said that “A good set of bowels is worth more to a man than any quantity of brains” (Josh Billings, 1818-1885). Although this quotation was intended to be humorous there is some truth which is illustrated by the symptoms of the irritable bowel syndrome (IBS), which is a common functional bowel disorder, associated with abdominal pain, sensations of bloating and altered bowel habit. Much research effort has focused on the serotonin-modifying drugs to overcome dysfunction caused by perceived imbalance in either the amount of 5–HT released in the intestine or the expression of its receptors. This is because of the wealth of evidence showing that 5–HT alters the rate at which contents travel down the digestive tract and also the rate at which fluid is absorbed [31]. Consequently, 5–HT_3_ antagonists, 5–HT_4 _agonists and SSRI antidepressants have been the subject of intense investigation for the treatment of IBS [21,28]. For example, the 5–HT_4_ agonist, tegaserod, has been shown to reduce abdominal pain and give a degree of relief from other symptoms in patients with constipation-predominant IBS [61]. 5–HT_4_ receptors are present in several discrete tissue locations in the human colon. These include the mucosa where the response to 5–HT released by enterotoxins induces Cl^-^ secretion resulting in diarrhoea [19]. The 5–HT_4_ receptor is also present in the circular smooth muscle cells of the human colon [39]. 5–HT_4_ agonists induce relaxation and inhibition of spontaneous contractions by activating adenylyl cyclase to increase intracellular levels of cAMP [53,54]. Paradoxically, 5–HT_4_ receptors expressed by cholinergic neurones in the human colon oppose the effect of this inhibitory postsynaptic 5–HT_4_ population by enhancing acetylcholine release [47]. The 5–HT receptors of cholinergic nerve endings also function to enhance transmitter release to the longitudinal muscle bands (taenia coli) [66]. Consequently, it has been suggested that the effects of 5–HT itself and 5–HT_4_ agonists to facilitate colonic propulsion are partly achieved by a coordinated combination of circular muscle relaxation and longitudinal muscle contraction [66]. Another less well established location of the 5–HT_4_ receptor in the human colon is on sensory nerve endings, where its function may be to increase sensory perceptions arising from the abdomen leading to altered motility patterns. A recent clinical study lends some support to this hypothesis, which showed that IBS patients have significantly lower perception and defecation thresholds to rectal thermal and pressure stimuli compared to age and gender matched control subjects [49]. The human small intestine and colon express various h5–HT_4_ splice variants (see Table **1**, [13,15,56]). It is possible that different 5–HT_4_ receptor splice variants are expressed in the various locations and so contribute to the regulation of bowel functions in different ways. However, there is a lack of quantitative data for 5–HT_4_ receptor splice variant expression relative to the colon to substantiate such speculations.

### 5–HT_7_ Receptors

5–HT_7_ receptors have also been identified in the circular muscle of the human colon, but as yet not in nerves [39,65]. All 5–HT_7_ receptor splice variants are expressed in the human small intestine and colon including a certain amount of the h5–HT_7(d+5) _fragment [43]. 

The patent literature also covers the potential use of novel full and partial 5–HT_7_ receptor agonists. The proposed applications are for the treatments of depression, anxiety and eating disorders, correction of circadian rhythm and migraine. As with the 5–HT_4_ ligands, none are suggested to be splice variant-selective. These applications are well-founded given the widespread distribution of the 5–HT_7_ receptor in the CNS and accumulating evidence for its functional effects. This includes the findings that some antidepressants, such as amitriptyline, have relatively high affinities at the 5–HT_7_ receptor (see [48,50,73] for reviews)**. **The 5–HT_7_ receptor was originally discovered in the CNS where particularly high expression was found in the thalamus and hypothalamus. Functional studies of these areas led to the suggestion that 5–HT_7_ receptors localised in the suprachiasmatic nuclei of the hypothalamus are involved in controlling circadian rhythm [51]. Although subsequent studies have generated some uncertainty, it has been established that the selective 5–HT_7_ receptor antagonist, SB-269970 reduces paradoxical sleep in rats [35,48]. It has also been suggested that the 5–HT_7_ receptor is involved in migraine (see [70] for review). Several lines of evidence support this, such as the findings that the receptor is expressed in cranial blood vessels of experimental animals and that 5–HT and 5–HT_7_-preferring agonists cause them to dilate. The transcript of the 5–HT_7_ receptor has been detected in human internal carotid and menigeal arteries, but functional evidence for its potential role in migraine is lacking. Other lines of evidence point to the involvement of the 5–HT_7_ receptor in hyperalgesic pain and neurogenic inflammation. The fact that prophylactic anti migraine 5–HT receptor antagonists have relatively high affinity for the 5–HT_7_ receptor further implicates this receptor in the abnormal vascular and neurogenic alterations that account for migraine headache. 

## CONCLUDING REMARKS

The intricate differences in structural forms of 5–HT_4_ and/or 5–HT_7_ splice variants suggest that they perform separate functions. This would have occurred over a long evolutionary period resulting in the refinement to the ancestral serotonin signalling system. Specific functions are also implied by the varying degrees of tissue-specific distribution of most splice variants and that such functions are important to the organs in which they are expressed (Table **1**) and the disease state of the tissue. Refinement of function is conferred by the C terminal tails of the splice variants which allow them to interact with different GIPs, GRKs and even different G proteins. To date, the established net outcome of splice variant-specific interactions within the cell determine factors such as the rate, duration and intensity of the response to 5–HT.

Although it has been shown that some splice variants have different sensitivities to ligands, it has only been demonstrated with a small number of agonists. It remains a considerable challenge to identify and develop splice variant-selective drugs (no antagonists have yet been identified). However, the pressure to do so may increase as a result of the recent voluntary restriction of tegaserod to Special Access use only. This was necessary as IBS patients have been shown to experience a higher chance of cardiovascular events, such as heart attack, stroke, or severe heart-related chest pain. This predictable problem could be avoided if it were possible to discover an agonist with reasonable selectivity for a splice variant(s) present in the intestines, but not in the heart (e.g. 5–HT_4(d or f)_). Future drug discovery projects may also turn to the GIPs and GRKs in order to achieve tissue-specific effects.

## Figures and Tables

**Fig. (3):**
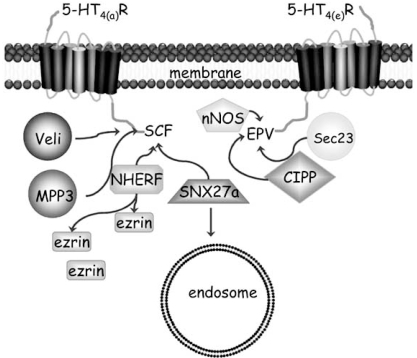
Example of PDZ proteins interacting with mouse 5–HT_4_ receptor splice variants (a) and (e). The PDZ proteins, Veli, MPP3, NHERF and SNX27a interact with the extreme C-tail of 5–HT_4(a)_ which contains the amino acids SCF. NHERF interacts with the scaffold protein ezrin and is involved in localising the 5–HT_4(a)_ splice variant to specific parts of the membrane and SNX27a is involved at a later stage in assisting the receptor to be targeted to endosomes for desensitisation. The 5 HT_4(e)_ receptor splice variant associates with nNOS, Sec23 and CIPP (another scaffolding protein). Ar-rows indicate protein interactions. For further details see text.

**Table 1.** Distribution of Human 5–HT_4_ and 5–HT_7_ Receptor Splice Variants

Tissue	5–HT_4_ Splice Variant	5–HT_7_ Splice Variant
Heart	atrium: ; a, b, c, g, n, i ventricle: a, b, g, i	Total: a, b, d, d+5
Brain	a, b, c, g, long c, n, e, f, i	a,b, d
Kidney	a, b	a, b, d
Bladder	a	
Spleen		a, b, d
Lung		a, b, d
Oesophagus	a, b, n	
Stomach	a, b, long c	
Ileum	a, b, c, d, g, e, f, i	a, b, d, d+5
Colon	a, b, long c, d, g, e, f, i	a, b, d, d+5

Summarised from the following literature for 5–HT_4_ receptors: [6,13,15,20,56,58,74] and 5 HT_7_ receptors: [36,43]
